# Shifting memories

**DOI:** 10.7554/eLife.30774

**Published:** 2017-09-11

**Authors:** Hong-Viet V Ngo, Bernhard P Staresina

**Affiliations:** School of PsychologyUniversity of BirminghamBirminghamUnited Kingdom

**Keywords:** Motor learning, sleep, consolidation, fMRI, striatum, neuroplasticity, Human

## Abstract

A region of the brain called the putamen has a central role in procedural memory consolidation during sleep.

**Related research article** Vahdat S, Fogel S, Benali H, Doyon J. 2017. Network-wide reorganization of procedural memory during NREM sleep revealed by fMRI. *eLife* **6**:e24987. doi: 10.7554/eLife.24987

As easy as riding a bicycle or driving a car might seem now, we probably all remember the blood, sweat and tears we spilled during our very first attempts. So how did we overcome those initial hurdles? While repeated practice undoubtedly plays a big part, sleep is also thought to strengthen such procedural memories (and also other types of memories) over time (Doyon and Benali, 2005; Maquet, 2001). Indeed, the lack of distractions while we are asleep provides an ideal opportunity for the brain to process and reinforce memories laid down during the day, also known as memory consolidation.

According to the concept of ‘systems consolidation’, new experiences or skills are gradually moved from regions of the brain involved in learning to a more widespread network that allows for long-term storage and integration with previous memories (Frankland and Bontempi, 2005). However, direct evidence for such a process is scarce, partly because it is difficult to monitor dynamic neural processes in the sleeping brain. Now, in eLife, Julien Doyon of the University of Montreal and co-workers – including Shahabeddin Vahdat as first author, Stuart Fogel and Habib Benali – report compelling new insights into the consolidation of procedural memories during sleep (Vahdat et al., 2017). The experiments involved the use of functional magnetic resonance imaging (fMRI) to monitor the activity of different brain regions as the participants in the study slept while, simultaneously, using electroencephalography (EEG) to relate any changes in brain activity to particular stages of sleep.

To assess the influence of sleep on memory consolidation, Vahdat et al. asked the participants in the study to perform a simple motor learning task in which they had to tap their fingers in a particular sequence, similar to learning a new melody on the piano (Debas et al., 2010). The participants learned the task in the evening and were tested again the following morning. In between they spent the first 2.5 hours of sleep in the fMRI machine. In a control experiment that took place on a separate occasion, participants performed a task in which they rhythmically, but without a particular sequence, had to move all their fingers (similar to repetitively tapping a drum).

Vahdat et al. compared the fMRI scans recorded during the two tasks and discovered that, contrary to the control task, the brain regions involved in the learning task differed markedly between the morning and the evening session. In the evening session, activity was mainly seen in the brain’s outer layer, the cortex, but after a night’s sleep, new areas located deeper within the brain were active. When analyzing the EEG data, the researchers observed that this gradual shift began during a stage of sleep called nREM (non-rapid eye movement) sleep. Researchers have previously linked this stage of sleep, which accounts for about two thirds of a typical night's sleep, to memory consolidation (Rasch and Born, 2013). Together, these findings suggest that the consolidation of procedural memory is a dynamic process that relies on the reorganization within the relevant networks in the brain during sleep.

Vahdat et al. wanted to know what happens in the sleeping brain during these reorganization processes. Using a series of elegant analyses, they were able to pinpoint that a region called the putamen acted as a hub for the shifts they observed. The putamen is a key region of the brain’s motor network, and while the participants slept, the connections between the putamen and other brain regions increased (Figure 1). What’s more, the intensity of the connections also predicted how well a participant would perform the learning task the next morning. In other words, the stronger the putamen interacted with other brain regions during sleep, the better the participant would be at performing the finger-tapping task the next morning.

**Figure 1.**
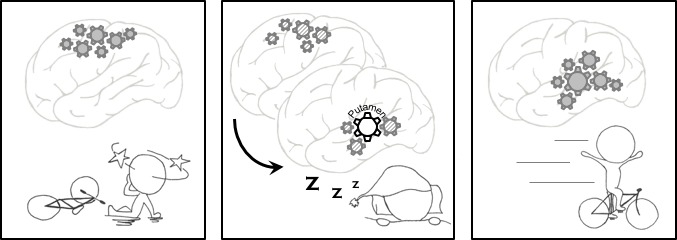
Memory consolidation. Left: The procedural memory we rely on to learn and perform certain tasks, such as cycling, improves with practice and sleep. Repeated practice activates the relevant neurons and strengthens the connections between them. Middle: During the night, these neurons are reactivated, and when we enter a stage of sleep called nREM sleep, a region of the brain called the putamen oversees the transfer of these patterns of activity from the cortex (the outer layer of the brain) to regions located deeper within the brain. Right: Vahdat et al. showed that stronger connections between the putamen and other brain regions resulted in the participants in their experiments performing better in a learning task the following morning.

IMAGE CREDIT: H-V V Ngo.

Altogether, Vahdat et al. provide compelling evidence that sleep helps to reorganize the brain networks underlying procedural memory, and highlight the importance of the putamen in orchestrating these consolidation processes. The findings open exciting avenues for future research. It would be interesting to see how other sleep stages, in particular REM sleep, contribute to the consolidation of memories (Walker and Stickgold, 2006). EEG recordings make it fairly easy to distinguish nREM sleep from other stages, and to directly link particular hallmarks of sleep to changes in brain dynamics (Fogel et al., 2017; Latchoumane et al., 2017). The challenge here lies mainly in keeping participants comfortable enough in a brain scanner to reach this late stage of sleep.

Finally, the current findings raise the question of whether other types of memories use comparable mechanisms and if other brain regions could have a comparable role to the putamen for them (Eichenbaum et al., 2007). These and related questions are worth pursuing, and will undoubtedly enhance our understanding of the fascinating relationship between sleep and memory.
